# Reliability and responsiveness of the Swedish short Hip‐RSI

**DOI:** 10.1002/jeo2.12029

**Published:** 2024-05-15

**Authors:** Tobias Wörner, Mikael Sansone, Anders Stålman, Frida Eek

**Affiliations:** ^1^ Department of Health Sciences Lund University Lund Sweden; ^2^ Department of Molecular Medicine and Surgery, Stockholm Sports Trauma Research Center Karolinska Institutet Stockholm Sweden; ^3^ Gothenburg Sports Trauma Research Center, Institute of Cinical Sciences, Sahlgrenska Academy University of Gothenburg Gothenburg Sweden

**Keywords:** hip arthroscopy, psychological readiness, return to sports

## Abstract

**Purpose:**

The aim of this study was to examine test–retest reliability and responsiveness of the short version (6‐item) Hip Return to Sport after Injury (Hip‐RSI) scale in patients following hip arthroscopy.

**Methods:**

The study included 100 hip arthroscopy patients responding to a digital survey including the short version (6‐item) Hip‐RSI, International Hip Outcome Tool (short version) (iHOT‐12) and RTS status 3, 6 and 9 months following surgery. The Hip‐RSI was administered twice at 3‐month follow‐up. Test–retest reliability was evaluated using intraclass correlation coefficients. Responsiveness was tested by correlations between changes in Hip‐RSI and iHOT‐12 scores and by comparing change in Hip‐RSI scores of patients who progressed on the return to sport (RTS) continuum (from return to any sport to return to performance) to patients who did not, using independent samples *t*‐tests.

**Results:**

Hip‐RSI was found to have excellent test–retest reliability on the individual (intraclass correlation coefficient, ICC [95% confidence interval, CI]: 0.90 [0.83–0.94]) and group level (ICC [95% CI]: 0.95 [0.91–0.97]) with a standard error of measurement of 5.53 and smallest detectable change of 15.3 on the individual and 2.2 on the group level. Hip‐RSI was found responsive to change through positive correlations of changes in scores with changes in iHOT‐12 scores from 3 to 6 months (*r* [95% CI]: 0.51 [0.35–0.65]; *p* < 0.001) and from 3 to 9 months following arthroscopy (*r* [95% CI]: 0.61 [0.57–0.79); *p* < 0.001). Further responsiveness was shown by significant mean changes in scores among patients that progressed on the RTS‐continuum (3–6 months: 8.6 [95% CI: 3.8– 13.5); 3–9 months: 12.6 [5.6–19.7]).

**Conclusion:**

The short version (6‐item) Hip‐RSI demonstrated excellent test–retest reliability and responsiveness to change in the evaluation of psychological readiness to RTS following hip arthroscopy.

**Level of Evidence:**

Level II.

**Abbreviations**
ACL: anterior cruciate ligament
ACL‐RSI: Anterior Cruciate Ligament‐Return to Sport after injury scale
FAIS: femoroacetabular impingement syndrome
Hip‐RSI: Hip Return to Sport after Injury scale
HSAS: Hip Sports Activity Scale
ICC: intraclass correlation coefficient
iHOT‐12: International Hip Outcome Tool (short version)
PROM: patient‐reported outcome measure
RTS: return to sport
SD: standard deviation
SDC: smallest detectable change
SEM: standard error of measurement
SRM: standardised response mean

## INTRODUCTION

Psychological readiness is recommended to be evaluated as part of the return to sport (RTS) decision following injury [1]. In the RTS process following hip arthroscopy for femoroacetabular impingement (FAI) syndrome, clinicians consider psychological readiness to be one of the most decisive factors in the RTS process [22]. In recent years, the Hip Return to Sport after Injury (RSI) scale has been introduced as a valid patient‐reported outcome measure (PROM) for the assessment of psychological readiness in patients following hip arthroscopy [7, 23].

The Hip‐RSI is based on the anterior cruciate ligament (ACL) RSI, which measures psychological readiness to RTS in patients following ACL reconstruction by assessing (a) the athlete's emotions, (b) confidence in performance and (c) risk appraisal through 12 visual analogue scale items [21]. A short version (6 items) of the ACL‐RSI has been shown to be valid, reliable and responsive to change in relation to knee‐related quality of life [19, 20].

The full version (12‐item) Hip‐RSI has been shown to have adequate construct validity in two separate studies [7, 23], but only one of these studies [23] evaluated content validity by involving patients and clinicians as recommended by the COSMIN guidelines [13]. Based on patients' and clinicians' responses, an item‐reduced version (6 items) of the Hip‐RSI was presented and demonstrated construct and content validity for the assessment of psychological readiness to RTS in patients following hip arthroscopy [23]. However, other psychometric properties of the short version (6‐item) Hip‐RSI such as test–retest reliability and responsiveness have not been investigated.

The aim of this study is to assess the test–retest reliability and responsiveness of the Swedish short (6‐item) version Hip‐RSI in patients following hip arthroscopy for FAI syndrome.

## METHODS

This psychometric study was planned and reported with consideration of the COSMIN guidelines [10]. All participants provided written informed consent prior to inclusion into the study, which was approved by the Swedish ethical review authority (Dnr 2020‐03206).

### Participants

Participants were recruited within a prospective study, investigating RTS following hip arthroscopy for FAI syndrome. The first 100 consecutive patients scheduled for hip arthroscopy at two surgical centres between February 2021 and August 2022 who had fully evaluable data were included in this study. Exclusion criteria for this study were radiological evidence of osteoarthritis, previous hip arthroscopy on the same hip and concurrent injuries with the potential to influence RTS.

### Data collection

Data were collected via web‐based surveys at baseline, 3 months (plus 2 weeks afterwards), 6 months and 9 months following surgery. The baseline survey was administered prior to surgery and included questions regarding basic demographic information such as gender, age, height and weight. Participants were also asked to report previous and current activity levels (Hip Sports Activity Scale [11]) as well as symptom duration. At 3, 6 and 9 months following surgery, participants were asked to respond to the short (6‐item) version of the Swedish Hip‐RSI (from here on referred to as short Hip‐RSI) [23], report their hip‐related quality of life on the short version of the International Hip Outcome Tool (iHOT‐12) [4] as well as their RTS status. RTS status was assessed by an ordinal question asking participants if they either (1) returned to any kind of sport, (2) returned to a different sport than the previous, (3) returned to the previous sport but not to the same performance level or (4) returned to the previous sport at previous performance level. For the purpose of assessing test–retest reliability, participants were asked to respond to the short Hip‐RSI again 1–2 weeks after the 3‐month follow‐up. The period of 1–2 weeks was considered long enough to minimise the risk for participants to recall their previous responses and short enough to minimise the risk for changes in readiness to RTS. The stability of the participants' psychological readiness to participate in sports was assessed by asking them whether their readiness has (a) increased, (b) remained stable or (c) decreased since they responded to the short Hip‐RSI 1–2 weeks ago.

### Statistical analysis

Test–retest reliability was analysed by calculating (two‐way mixed model with absolute agreement) intraclass coefficients (ICCs) 1 (single measures) and ICC 2 (average measures) with 95% confidence interval (CI). ICCs were calculated for the subgroup of participants reporting unchanged status in the construct to be measured. A paired *t*‐test was performed to test for systematic differences between mean scores (measured 2 weeks apart). We expected there to be no statistically significant difference between test and retest and adequate reliability with an ICC of ≥0.7 [15]. Standard error of measurement (SEM) was calculated according to the formula SD × √(1 − ICC), with standard deviation (SD) for the scale among the included sample at 3 months, and ICC 2. Smallest detectable change (SDC) was calculated on the individual and group level according to the formula 1.96 × √2 × SEM on the individual level and 1.96 × √2 × (SEM/√*n*) on the group level [2, 3].

Responsiveness was assessed through a construct approach [9]. Associations between the change in short Hip‐RSI scores and the change in iHOT‐12 from 3 to 6 and from 3 to 9 months following arthroscopy were assessed by Pearson's correlation coefficients. A change in total short Hip‐RSI score with a positive correlation of *r* > 0.5 with a change in iHOT‐12 was considered sufficient responsiveness [9]. Participants were categorised into either improvement in RTS status (at least one step increase from, for example, ‘returned to any kind of sport’ to ‘returned to previous sport’) or no improvement (no change or reduction) between 3 and 6 months and between 3 and 9 months. Changes in short Hip‐RSI were analysed for each group with a paired samples *t*‐test, and standardised response mean (SRM) presented according to Cohen's *d* (mean change/sample SD of mean change). The changes in short Hip‐RSI were compared between groups with improved versus not improved level of RTS with an independent samples *t*‐test. *p* < 0.05 was considered statistically significant.

## RESULTS

Between May 2021 and November 2022, the first 100 consecutive participants who had surgery at participating clinics and responded to the baseline survey, the 3‐month follow‐up and the test–retest survey were included in the study. Test–retest reliability was evaluated in 48 of the 100 participants who reported unchanged status in the construct to be measured while the other 52 (47 reported improved status, four reported worse status and one responded too late; >3 weeks after 3‐month follow‐up) were excluded from the analysis. Assessment of responsiveness was based on 93 of the 100 participants who answered the 6‐month follow‐up and 96 of 100 participants who responded to the 9‐month follow‐up. The demographic information of the final sample is summarised in Table 1.

**Table 1:** Demographic information (*n* = 100).

Sex (%)	
Female	26
Male	74
Age at time of surgery (years), mean (SD)	31.4 (9.5)
Height (cm), mean (SD)	179 (9.2)
Weight (kg), mean (SD)	80 (12.9)
HSAS, median (IQR)	
In adolescence	7 (5–8)
Prior to symptoms	7 (5–8)
Prior to hip arthroscopy	3 (2–5)
Symptom duration (*n*/%)	
6–12 months	9
1–2 years	29
>2 years	62

Abbreviations: HSAS, Hip Sports Activity Scale; IQR, interquartile range; SD, standard deviation.

### Test–retest reliability

Patients answered the retest at a mean time of 10 days (SD 4; range: 4–21). In accordance with our a priori expectations, we found excellent test–retest reliability with an ICC 1 (single measures) of 0.90 (95% CI: 0.83–0.94) and ICC 2 (average measures) of 0.95 (95% CI: 0.91–0.97). Furthermore, we found no systematic differences between test and retest scores on the group level (mean difference: 0.12; 95% CI: −3.03 to 3.27; *p* = 0.939). The SEM for the scale was 5.53. The SDC for the scale was found to be 15.3 on the individual and 2.2 on the group level.

### Responsiveness

As expected a priori, we found changes in short Hip‐RSI scores to be positively correlated to changes in iHOT‐12 scores between 3 and 6 months (*r* [95% CI]: 0.51 [0.35–0.65]; *p* < 0.001) and between 3 and 9 months (*r* [95% CI]: 0.61 [0.57–0.79]; *p* < 0.001) postoperatively. Furthermore, short Hip‐RSI scores increased significantly for patients that increased their RTS status from 3 to 6 months (*n* = 40; mean change: 8.6, 95% CI: 3.8–13.5; SRM: 0.57, 95% CI: 0.23– 0.90; *p* < 0.001) and from 3 to 9 months following surgery (*n* = 45; mean change: 12.6, 95% CI: 5.6–19.7; SRM: 0.54, 95% CI: 0.22–0.85; *p* < 0.001). No significant changes were found among patients with no increase in RTS status from 3 to 6 months (*n* = 53: mean change: 0.52, 95% CI: −4.9 to 6.0; SRM: 0.03, 95% CI: −0.24 to 0.30; *p* = 0.848) or from 3 to 9 months (*n* = 51; mean change: −3.6, 95% CI: −9.1 to 1.97; SRM: −0.18, 95% CI: −0.46 to 0.10; *p* = 0.20). The change was significantly larger in the group with improved RTS status from 3 to 6 months (*p* = 0.033) and from 3 to 9 months (*p* < 0.001) compared with the group with no improvement in RTS status.

## DISCUSSION

This study complements the previous psychometric evaluation of the short Hip‐RSI, which has found the scale to be a valid PROM in the evaluation of patients following hip arthroscopy [23]. This current psychometric evaluation found the short Hip‐RSI to be reliable and responsive to change in patients following hip arthroscopy.

### Test–retest reliability

In accordance with the a priori hypothesis, the short Hip‐RSI demonstrated excellent test–retest reliability. In comparison to the longer, 12‐item version of the Hip‐RSI [7], the short version demonstrated higher ICCs as well as smaller SEM and SDCs. Hence, clinicians can be confident that the short Hip‐RSI provides consistent scores of psychological readiness to RTS in patients following hip arthroscopy, and the scale may be used to evaluate progress prospectively. When interpreting the scale, clinicians should be aware of the measurement error of about 5% (5.5 points on a scale of 0–100). A real change in scores can be considered if an individual patient presents with a change in scores of at least 15% and/or a group of patients presents with changes of at least 2.2%. In comparison to other PROMS, recommended to be used in patients following hip arthroscopy [5], measurement error and SDC are similar [8, 16]. However, less than half of the patients in this study were included in the test–retest analysis because the other half reported changes in psychological readiness to RTS within the 10 days between test and retest measurement. Psychometric evaluation of the short version of the ACL‐RSI indicates similar instability in psychological readiness [18], implying this observation to be valid across different patient groups. Hence, clinicians should keep in mind that psychological readiness may fluctuate over time, irrespective of actual changes in the joint‐specific condition of patients.

### Responsiveness

In accordance with the a priori expectation on the direction and strength of the correlation in changes between the short Hip‐RSI and iHOT‐12, the Hip‐RSI was found to be responsive to change. Previous research illustrates general patterns across different domains of self‐reported hip function where patients report the highest degree of disability in the domains related to sport and quality of life—both before and after surgical treatment [17]. The results of this study, showing positive correlations between changes in short Hip‐RSI and iHOT‐12 (measuring hip‐related quality of life) scores, further illustrate the relevance of the ability to participate in sports for this group of patients. Further evidence of short Hip‐RSI's responsiveness to change was provided by significant increases in scores among participants who improved their status on the RTS continuum between 3 and 6 months and between 3 and 9 months following surgery. The observed changes were above the SDC for evaluation of patient groups but below the SDC for individual patient evaluation. Hence, while changes in short Hip‐RSI scores have to be interpreted with caution in the evaluation of individual patients, the scale can be used with confidence in the evaluation of patient groups.

### Methodological considerations

The current study complements a previous evaluation of psychometric properties of the short (6‐item) version Hip‐RSI [23]. Readers are therefore recommended to review these studies in combination to assure a full overview of short Hip‐RSI's psychometric properties. All participants in this study have undergone hip arthroscopy for FAI syndrome, and results can therefore only be generalised to this patient group. However, patients with FAI syndrome [14], patients with other hip‐related causes of groin pain such as hip dysplasia [6] and patients with clinical entities such as adductor‐related groin pain [12] have similar patterns of patient‐reported function. Hence, the short Hip‐RSI may also be an appropriate measure for other diagnoses than only FAI syndrome, which should be investigated in future studies. According to the COSMIN study design checklist, the sample size of this study is adequate to measure test–retest reliability and responsiveness.

## CONCLUSION

The short (6‐item) version of the Hip‐RSI is a reliable and responsive PROM for the assessment of psychological readiness to RTS in patients following hip arthroscopy.

## AUTHOR CONTRIBUTIONS

All authors contributed to the conception of the study. Tobias Wörner was responsible for the recruitment of participants and collection of data. Frida Eek performed the statistical analysis, and data were interpreted in collaboration with Tobias Wörner. Tobias Wörner wrote the manuscript, which was critically reviewed and approved by all authors.

## CONFLICT OF INTEREST STATEMENT

The authors declare no conflict of interest.

## ETHICS STATEMENT

The study was approved by the Ethics committee at Lunds University (Dnr 2020‐03206). All participants provided written informed consent prior to inclusion into the study.

## Data Availability

The data that support the findings of this study are available from the corresponding author upon reasonable request.
